# Japanese Encephalitis Outbreak in Young Adults of Bastar District in Chhattisgarh: A Short Observational Study

**DOI:** 10.7759/cureus.55939

**Published:** 2024-03-11

**Authors:** Jaishriram Rathored, Rani Soni, Sandesh Shende, Debashish Samal

**Affiliations:** 1 Department of School of Allied Sciences, Central Research Laboratory and Molecular Diagnostics, Datta Meghe Institute of Higher Education & Research, Wardha, IND; 2 Department of Microbiology, Late Baliram Kashyap Memorial Government Medical College, Jagdalpur, IND

**Keywords:** monocyte chemotactic protein, tumor necrosis factor α, central nervous system, immunoglobulin, acute encephalitis syndrome, japanese encephalitis virus

## Abstract

Background

Instant infections in children due to acute encephalitis syndrome (AES) were reported in a tribal district of Bastar in Chattisgarh, India, between August 2018 and August 2019.

Objective

The study was conducted to explore the possibility of a viral cause indicating an outbreak.

Methods

Clinical surveys and serological investigation tests were conducted to identify the viral etiology. The Bastar area in Chhattisgarh reported factors such as paddy fields near homes, a high pig-to-cattle ratio, a significant presence of *Culex vishnui* mosquitoes, low socioeconomic status, and a lack of health awareness among the tribal people.

Result

This study, conducted at the Late Baliram Kashyap Memorial Government Medical College in Jagdalpur, Bastar, Chhattisgarh, India, analyzed 128 samples from fever cases out of 213 patients visiting the Japanese encephalitis virus (JEV) testing center. Among these samples, 71 cases exhibited AES, and subsequent JEV IgM ELISA testing identified 18 cases as JEV-positive, signifying recent JEV infections. Notably, the overwhelming majority (94.44%) of JEV-positive patients were under 16 years old, highlighting the heightened vulnerability of children to JEV illness in the Bastar region. Although male patients accounted for 61.11% of the JEV-positive cases compared to 38.88% of female patients, statistical analysis revealed that this gender disparity was not statistically significant (p-value = 0.18).

Conclusion

The study emphasizes the significance of identifying the etiology and delivering evidence-based care to patients with AES. Improved diagnosis and management of AES may result from a greater comprehension of the advantages and disadvantages associated with the application and administration of common laboratory and diagnostic algorithms.

## Introduction

Japanese encephalitis (JE), a virus transmitted by mosquitoes, predominantly affects regions in the South, Southeast, East, and parts of the Pacific. Over the past few years, there have been numerous reports of JE infection in India, particularly in the North Eastern States, Bihar, Uttar Pradesh, West Bengal, and Andhra Pradesh. Additionally, the disease is spreading to less informed, non-endemic regions of the country [1]. This poses significant public health challenges in endemic areas, with potentially severe consequences, including acute encephalitis syndrome (AES), characterized by high fever and CNS involvement [2-4].

The National Vector Borne Disease Control Program in India plays a pivotal role in JE control and policy formulation, providing diagnostic kits and vaccinations to mitigate its impact. Despite the low incidence of JE among adults due to prior exposure [5,6], the virus remains a concern, with approximately 1 in 300 infected individuals developing serious symptoms [7,8].

Transmission of JE primarily occurs through* Culex* mosquitoes, with species such as *Culex tritaeniorhynchus* responsible for human transmission in India [9-11]. Rice cultivation areas with abundant rainfall, pig farming [12], and habitats frequented by migratory birds serve as favorable environments for JE transmission, highlighting the ecological complexity of the disease [13,14].

The majority of JE cases resolve in five to seven days during their incubation period, which can last anywhere from five to 14 days [15,16]. However, individuals presenting with AES symptoms undergo serological testing for JE confirmation, with IgM antibodies [17,18] serving as the primary indicator of recent JEV infection [19,20]. Blood samples were tested using the JE virus (JEV) IgM (ELISA kit, National Institute of Virology (NIV), Pune).

## Materials and methods

Study setting

The hospitalized patients in the cross-sectional study had elevated fever and AES. The current study, conducted under the direction of the NIV, took place in the Department of Microbiology (VRDL) at the Late Baliram Kashyap Memorial Government Medical College in Jagdalpur, Bastar, Chhattisgarh, India.

Sample size

As this is an outbreak study, the sample size was not predetermined; instead, all samples were collected between August 2018 and August 2019.

Inclusion and exclusion criteria

The study enrolled individuals aged 18-65 who resided in a Chhattisgarh district and had been admitted to the hospital with AES and a high fever. Exclusion criteria included individuals who had left the affected districts within a specific timeframe. Additionally, those with HIV and comorbid conditions such as diabetes and seizures were not included in the study. Patients who tested positive for the malaria parasite were also excluded through rapid antigen detection and microscopy.

Data collection and population

The present investigation focused on patients admitted to the Late Baliram Kashyap Memorial Government Medical College with a high fever and AES. AES, characterized by a sudden fever and neurological symptoms such as delirium, coma, mental confusion, and disorientation, is caused by the JE virus, a zoonotic pathogen. The virus is primarily transmitted to humans through the Vishnu group *Culex* vector, with animals, birds, and pigs serving as intermediate hosts. Birds belonging to the Ardeidae family, including pond herons and cattle egrets, are common carriers. The investigation was conducted under the direction of the NIV at the Department of Microbiology (VRDL) of the Late Baliram Kashyap Memorial Government Medical College. Patients who tested positive for the malaria parasite through rapid antigen detection and microscopy, based on specific criteria, were excluded. Between August 2018 and August 2019, a total of 213 patients were admitted to the Late Baliram Kashyap Memorial Government Medical College. Among them, 128 had positive fever cases, and 71 received an AES diagnosis. All 71 AES samples underwent testing using JEV-IgM Capture ELISA, leading to the identification of 18 reactive samples. The study region, Jagdalpur, situated in the Bastar district of Chhattisgarh, India, has an average elevation of 552 meters (1,811 feet) above sea level, with coordinates of approximately 19.07° North latitude and 82.03° East longitude. The cross-sectional analysis based on laboratory work was carried out at the NIV of the Late Baliram Kashyap Memorial Government Medical College.

Laboratory analysis

The NIV provided a kit for the JEV IgM ELISA test, which was used in accordance with the manufacturer’s instructions. In short, separate microwells were filled with 50 microliters of 1:100 diluted serum from each sample, a positive control, and a negative control. The microwells were then incubated for an hour at 37°C. The wells were cleaned, then the antigen was added and incubated again. Then, monoclonal antibodies, TMB substrate, and horseradish peroxide were added. Absorbance measurements were performed at 415 and 630 nanometer wavelengths using an ELISA reader. Each sample’s 50 microliters of diluted serum were placed in a microwell, along with 50 microliters of positive and negative controls in separate wells. The plate was then incubated for one hour at 37°C. Following incubation, the wells were rinsed five times with washing buffer, antigen was added, and the mixture was incubated for an hour at 37°C. Next, the wells were rinsed five times with monoclonal antibodies, horseradish peroxide was repeated for incubation, and TMB substrate was added. Finally, absorbance measurements were obtained using an ELISA reader at wavelengths of 415 and 630 nanometers [21].

Statistical analysis

The results were analyzed for a descriptive study considering the frequency and percentage of outcome evaluation generated for the JE incidence using IBM SPSS Statistics for Windows, Version 25.0 (Released 2017; IBM Corp., Armonk, NY, USA). The incidence of JE by gender among males and females and age group distribution was calculated using Chi-square analysis and found to be significant for gender as a p-value ≤ 0.05.

## Results

A total of 128 samples from fever cases were received from the 213 patients who attended the JEV test and testing center at the Late Baliram Kashyap Memorial Government Medical College (Table 1). AES was present in 71 samples, and these 71 samples were tested using JEV IgM CAPTURE ELISA. Reactive tests were performed on 18 samples. Nineteen of the 18 patients (94.44%) are younger than 16. This causes JEV illness to impact children in this Bastar region.

**Table 1:** Total number of JEV cases AES, acute encephalitis syndrome; JE, Japanese encephalitis; JEV, JE virus

S. no	JEV	Total cases	Fever cases	JE with AES positive	Positive patients
1	JEV	213	128	71	18

Male patients are more compared to female patients (Table 2), although this difference is not statistically significant. Before the completion of the serological testing, two patients passed away.

**Table 2:** Number of cases between males and females from different age groups

Age group	Number of cases	Male	Female
0-6 years	9	5	4
5-11 years	3	1	2
10-16 years	5	4	1
More than 15 years	1	1	0
Total	18	11	7

The study presents a detailed analysis of the demographic and virological characteristics of positive patients in Table 3, categorized by gender. One male patient and two female patients made up the participants; the patients’ ages ranged from one to 20 years for the males and from one to 16 years for the females. Male patients had a median age of 10 years (range: eight to 12 years), while female patients had a median age of eight years (range: six to 10 years). Clinically, the viral load levels in male and female patients were comparable, ranging from 10 to 40 log_10_ copies/ml. Serum levels were also measured, with a mean of 5.57 nmol/l for patients who were male and 6.08 nmol/l for patients who were female. A p-value of 0.73 indicated that there were no significant differences in serum levels between the genders, and a p-value of 0.51 indicated that there were no significant differences in viral load.

**Table 3:** Viral load and demographics of patients who tested positive for JE JE, Japanese encephalitis

Parameters	Male	Female
Age	(1-20)	(1-16)
Median age	10 (8-12)	8 (6-10)
Viral load	10-40 log_10_ copies /ml	10-40 log_10_ copies/ml
Serum	5.57 nmol/l	6.08 nmol/l
p-Value	0.73	0.51

## Discussion

Since Bastar, a remote tribal region of Chhattisgarh, is an AES endemic region, it is important to note that AES cases cannot be assumed to be solely JE cases because the majority of cases have non-JE origins. This raises concerns because more thorough research is required to determine the multifactorial etiology of non-JE cases so that appropriate measures can be taken to reduce the disease burden in this region of the state. Humans contacted JEV through mosquito bites from infected *Culex *species mosquitoes, primarily *C. tritaeniorhynchus*. Once infected, humans do not become sufficiently viremia positive to infect mosquitoes that are feeding. The virus is spread through the enzootic cycle, which involves mosquitoes, pigs, and/or water birds. Because people live closer to these vertebrate hosts in rural and peri-urban areas, these settings are where the disease is most common. The primary factors contributing to the incidence of AES in Bastar are the increased breeding and density of mosquitoes in monsoon-soaked paddy fields, the presence of pig farms near homes, and the lack of use of bed nets. Another factor is the fact that they frequently spend the day in the paddy fields, and day-biting* Culex* mosquitoes are the primary vector of JE. Rapid testing, such as sero-surveillance ELISA, can be employed to minimize the disease burden in terms of morbidity and mortality by taking appropriate action.

Our findings support the notion that men are more likely than women to encounter AES and JE, and other research has also revealed this difference in incidence. In our study, there is a greater proportion of male patients than female patients, despite the lack of statistical significance. The primary cause of the higher incidence among men is day-biting *Culex* mosquitoes, which are the primary vector of JE transmission and are more common in paddy fields where males are more likely than females to spend the day. Men had a higher percentage of cases (61.11%) compared to women (38.88%), according to JE. In line with findings from other Indian studies [22], our analysis did not reveal any statistically significant gender differences. A total of 110 of the 128 fever cases had JEV IgM non-reactive samples. Since JEV IgM cross-reacts with other flaviviruses, a different diagnostic tool was intended to be developed at the VRDL Jagdalpur.

Males in this area are more likely to work in rice fields and play outside, which increases their risk of getting bitten by *C. tritaeniorhynchus* mosquitoes. This could explain why men seem to prefer AES and JE. Since the vector usually breeds in the stagnant water of the cultivation fields, the majority of people in this age group are directly exposed to the vector. In our investigation, we found a correlation between JE infection and neighboring pigs, suggesting its possible role as a risk factor. The farming community faces a variety of risks, but the risks to production and the environment are more important. They frequently spend the day in the paddy fields, which is another factor. The main vector of JE is day-biting *Culex* mosquitoes. According to the current study, the most important factors influencing JE risk were being close to pigs, being close to paddy fields without using mosquito nets, and mosquito repellent. Huge populations of intermediate hosts, like pigs and cattle egrets, can be found in the Assamese paddy field ecosystems, and they are unquestionably essential to the spread of JE. There were documented cases of juvenile obesity in the age groups of 0-15 years (14.05%) and 16-30 years (16.22%) in our study. This could be because young people and children are more likely to be bitten by mosquitoes as a result of outdoor activities. This study bears similarities to earlier research conducted on the subcontinent [23].

It was found that children from rural areas were more impacted than those from urban areas. In most temperate regions of Asia, JEV is primarily spread during the warm season, when major epidemics can occur. Although transmission can occur year-round in tropical and subtropical regions, it often gains momentum in rice-growing regions during the pre-harvest and rainy seasons, resulting in a higher incidence rate in rural areas compared to urban areas. The higher incidence in rural areas was partly explained by the fact that individual preventive measures, like wearing long sleeves, using vaporizers and coils, and applying repellent to mosquitoes, were more widely adopted and used in urban areas. An additional significant factor contributing to the greater disease burden in rural areas is a lack of knowledge regarding vaccinations. The increased breeding and density of mosquitoes in monsoon-soaked paddy fields, the presence of pig farms near homes, and the lack of use of bed nets all lend credence to this conclusion. The majority of non-JE cases in this study’s area-wise distribution came from rural populations, with semi-urban and tea gardens coming in second and third, respectively. The majority of them work in farming and agriculture, so it is possible that this is the cause since they frequently interact with the different AES case vectors. Due to their frequent daytime activities in the paddy fields and the fact that day-biting *Culex *mosquitoes are the primary vector of JE, the majority of JE cases in the current study were reported from paddy fields. To put it succinctly, it could be managed through efficient surveillance systems, an integrated vector control strategy, pig vaccination, and segregation, as well as raising local population awareness of health issues. In order to lower the disease burden, more advanced molecular biological research plans are necessary to understand the pattern of AES cases in this region of the state. One of the study’s shortcomings is that, because only ELISA tests were carried out, additional molecular techniques are needed to confirm the fact. The three primary limitations of using an ELISA test alone for diagnosis are the prozone effect, the limit of detection and quantitation, and analytical noise. Furthermore, real-time PCR technology, which has high sensitivity and specificity, makes it possible to detect and quantify viral targets with greater accuracy from a variety of different clinical sample types. This is one of the most crucial ways to comprehend the genesis and patterns of AES and JE transmission in the area. Another limitation of the study was its examination of the JE/AES entomological outbreak, which made it impossible to plan for the number of man-hours spent. Given that this was an outbreak condition, there were a lot of other factors in the affected villages. As a result, tracking changes in density in the same villages was not feasible in all the districts of Chhattisgarh. This study’s other limitations include not accounting for the reasons behind the spatial heterogeneity of the JE incidence within climate subtypes and the lack of information on vectors or vertebrate density. Consequently, more research on the etiology of JEV transmission is required, including surveillance of the virus in hosts (pigs) and vectors, measurement of antibody levels in healthy individuals, and surveillance of diseases and vector mosquitoes.

## Conclusions

A major threat to India’s public health is the country’s propensity for predictable and frequent outbreaks of acute encephalitis in various regions. The nation of India’s frequent and predictable outbreaks of acute encephalitis pose a serious threat to public health. In addition to highlighting these populations’ vulnerability to newly emerging infections, the frequent occurrence of acute encephalitis outbreaks with high case fatality rates also emphasizes the need for enhanced health system capacity and focused response to these threats to protect the lives of socially vulnerable and impoverished populations. In areas where the JEV can spread, vaccination should be taken into consideration, even in cases where the number of confirmed cases is low. The frequent occurrence of acute encephalitis outbreaks with high case fatality rates not only highlights the susceptibility of these populations to emerging infections but also highlights the need for improved health system capacity and a targeted response to these threats in order to safeguard the lives of impoverished and socially vulnerable populations. The majority of AES cases lack an infectious aetiological agent, which poses a significant obstacle to effective prevention and management. A thorough understanding of the cause and mode of transmission is necessary for the development and successful application of prevention and management strategies. Second, a lack of sufficient adverse event surveillance makes it difficult to determine the exact disease burden, distribution, and trends in India. With an emphasis on the laboratory diagnostic component, enhanced AE surveillance within the broader vector-borne disease surveillance framework can help provide a more precise picture of the disease burden and epidemiology to support and direct programmatic interventions. Also, it is becoming more difficult to diagnose cases of encephalitis, encephalopathy, another neurological disorder, or any combination of these due to the growing clinical ambiguity. To create a case definition that makes sense in this scenario, which is not yet in use in India, carefully compiling information about the clinical presentation is crucial. This non-endemic area’s report on JE pointed to the need for public health awareness in areas where there is a chance of contracting JE infection due to environmental risk. Through early suspicion and investigation, this can reduce morbidity and mortality.

Establishing guidelines and raising public awareness of JE infection prevention and control would be easier with the identification of risk factors for the infection. These two approaches have the potential to lower the rate of JE infection in the relevant study area and help develop prompt public health interventions. There are JE vaccines that are both safe and effective in preventing illness. In all areas where JE is a recognized public health priority, implementing robust JE prevention and control measures, such as JE vaccination programs, in addition to bolstering surveillance and reporting systems, needs to be done. Where there is a favorable environment for the transmission of the JE virus, vaccination should be taken into consideration, even in cases where the number of confirmed cases is low. A decrease in the burden of JE disease from interventions other than human vaccination is not well supported by the data. Therefore, immunization of people ought to come before immunization of pigs and mosquito control techniques.
